# Flexible Strain Sensor Based on Carbon Black/Silver Nanoparticles Composite for Human Motion Detection

**DOI:** 10.3390/ma11101836

**Published:** 2018-09-27

**Authors:** Weiyi Zhang, Qiang Liu, Peng Chen

**Affiliations:** 1 School of Microelectronics, Tianjin University, Tianjin 300072, China; zhangweiyi@tju.edu.cn (W.Z.); pengchen@tju.edu.cn (P.C.); 2 Tianjin Key Laboratory of Imaging and Sensing Microelectronic Technology, Tianjin University, Tianjin 300072, China

**Keywords:** carbon black, silver nanoparticles, composite, flexible strain sensor, human motion detection

## Abstract

The demand for flexible and wearable electronic devices with excellent stretchability and sensitivity is increasing, especially for human motion detection. In this work, a simple, low-cost and convenient strategy has been employed to fabricate flexible strain sensor with a composite of carbon black and silver nanoparticles as sensing materials and thermoplastic polyurethane as matrix. The strain sensors thus prepared possesses high stretchability and good sensitivity (gauge factor of 21.12 at 100% tensile strain), excellent static (almost constant resistance variation under 50% strain for 600 s) and dynamic (100 cycles) stability. Compared with bare carbon black-based strain sensor, carbon black/silver nanoparticles composite-based strain sensor shows ~18 times improvement in sensitivity at 100% strain. In addition, we discuss the sensing mechanisms using the disconnection mechanism and tunneling effect which results in high sensitivity of the strain sensor. Due to its good strain-sensing performance, the developed strain sensor is promising in detecting various degrees of human motions such as finger bending, wrist rotation and elbow flexion.

## 1. Introduction

Among the sensors which have an electrical response to mechanical deformations, strain sensors have attracted considerable attention in many areas such as medical rehabilitation [1,2,3], sports performance monitoring [4,5], soft robots [6,7] and structural health monitoring [8,9]. As for the parameters of stretchable electric materials, stretchability, sensitivity, stability, fabrication cost, and simplicity are key factors determining the performance of strain sensors [10]. Even though traditional strain sensors made of metal foils and semiconductors are well-developed, their poor stretchability and high cost impose limitations as practical applications [11,12]. In recent years, there have been numerous efforts paid to enhance the performance of strain sensors with several alternative materials. Carbonaceous materials have been universally used in strain sensors because of their prominent electrical conductivity and mechanical properties [13]. The strain sensors made by fabricating the carbon nanotube (CNT) thin films on the flexible substrates have high stretchability, but these strain sensors suffered from low sensitivity [14]. In addition, the strain sensors fabricated by silver nanowires show relatively high sensitivity, which is suitable for human motion capturing. But the stretchability of these sensors is not ideal to monitor large-scale motions [10]. High-strain sensors with good sensitivity and stretchability have been reported by using graphene due to their flexibility and low sheet resistance [15,16,17]. Moreover, two-dimensional nanoflakes usually show a piezoresisitivity one order of magnitude higher than that of nanowires, since their electrical percolation network is largely susceptible to geometrical changes and discontinuities [15]. However, the relatively high fabrication cost for graphene and CNT is also a limitation on cost control of sensors based on these materials. Hence, achievement of strain sensors with high stretchability, high sensitivity and low cost is still a grand challenge [18].

Most widely employed strategies in preparation of flexible strain sensors are to use resistive-type sensors due to their relatively simple structure and fabrication process, as well as low energy consumption in operation [19,20]. Therefore, electrically conductive elastomer composites have been considered as satisfactory candidates to develop resistive-type flexible strain sensors owing to their excellent stretchability, intrinsically low modulus, cost-effectiveness and tunability of electrical properties [21,22]. Generally, conductive polymer composites (CPCs) for strain sensors are obtained by blending an insulating polymer matrix (thermoplastic or thermosetting plastic) with conductive fillers like carbon black (CB), carbon fibers or nanotubes, metallic particles or conductive polymers [23]. Thermoplastic polyurethane (TPU) can become the material of choice for making strain sensors due to the good combination of mechanical strength and elasticity, as well as the facile melt processability by comparison with other elastomers/rubbers [24]. There have been an increasing number of studies concerning the piezoresistive behavior of TPU nanocomposites with carbon-based nanofillers [17,25,26] or conductive metal nanoparticles [27]. They showed that the deformation of TPU nanocomposites under tensile strain could result in loss of contact between adjacent conductive fillers, leading to resistance-strain response. For the conductive filler, zero-dimensional (0D) carbon black (CB) is a suitable candidate because it possesses low aspect ratio, low cost (compared to other carbonaceous nanofillers such as graphene and CNT), and good conductivity. CB nanoparticles with a lower dimensionality have greater freedom to deform than one-dimensional (1D) CNT with high aspect ratio, leading to the easy damage of the impressible conductive paths under strain [13]. It is reported that due to the low aspect ratio of CB, more remarkable breakdown structure of the particles’ point-to-point conductive networks is observed during stretching and higher sensitivity can be achieved [28]. Besides, zero-dimensional CB powders could endow composites with excellent mechanical and electrical performance [29,30]. In addition, an increasing number of attentions have been attracted by silver nanoparticles (AgNPs) because the addition of AgNPs can contribute to form a synergic conductive network with other conductive materials to enhance the performance of the strain sensor utilizing the synergic effect [27,31] and propagate more microcracks in the conductive network under tensile strain. 

In this work, we propose a facile and low-cost strategy to fabricate a stretchable and sensitive strain sensor based on CB/AgNPs composite. This composite is fabricated through the modification and deposition process. As sensing element and filler, the composite is combined with TPU and then the mixture is casted to conductive film for preparation of strain sensor. The fabrication process is easily-operated and low-cost. Furthermore, the electrical property and sensitivity of the strain sensor can be controlled by adjusting filling level in TPU. The presence of AgNPs remarkably enhances the sensitivity compared with the bare CB-based strain sensor and the sensing mechanism is discussed according to the resistance model with junction identification. The electromechanical properties of a sensor such as static and dynamic characteristics of resistance variation are tested to verify the good sensitivity and stability. The capability of the CB/AgNPs composite-based strain sensor for detecting human motions like finger bending, wrist rotation and elbow flexion are also investigated.

## 2. Materials and Methods

### 2.1. Materials

Thermoplastic polyurethane (TPU, polyether resin Texin 985) was supplied by Bayer AG (Bayer Material-Science, Leverkusen, Germany). Graphitized carbon black nanopowder with a particle size of <500 nm was purchased from Sigma-Aldrich Co., Ltd. (Shanghai, China). Silver nitrate (AgNO_3_), trisodium citrate dihydrate (C_6_H_5_Na_3_O_7_·2H_2_O), anhydrous tetrahydrofuran (THF) and anhydrous ethanol were bought from Tianjin Kemiou Chemical Reagent Co., Ltd. (Tianjin, China). Sodium borohydride (NaBH_4_), liquid vinylpyrrolidone and benzoyl peroxide were obtained from Aladdin (Shanghai, China). All the chemical reagents were of analytical grade and were used as received without further purification.

### 2.2. Synthesis of Silver Nanoparticles (AgNPs) and Surface Modification of Carbon Black (CB)

**Figure 1:**
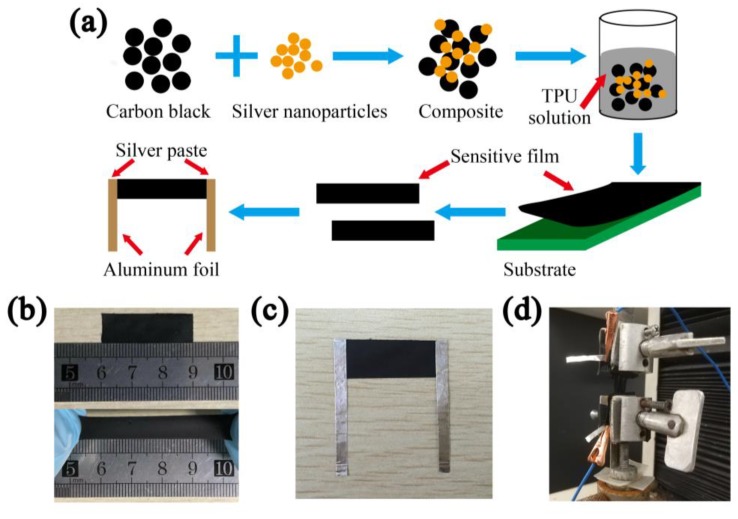
(**a**) Schematic illustration of the fabrication process of strain senor: fabrication of CB/AgNPs composite via surface modification process, mixing TPU and CB/AgNPs composite, scraping the mixture on the substrate to prepare conductive film, cutting the film into desire shape and electrodes attaching; photos of (**b**) the strain sensor with stretchable feature; (**c**) the sample of strain sensor with electrodes for ΔR/R_0_ vs. strain test and (**d**) the sensor clamped to the testing machine.

The overall fabrication procedure for CB/AgNPs composite-based strain sensor is schematically demonstrated in Figure 1a.

The method [32] based on the combination of the seed-mediated growth and the Lee-Meisel method by thermal reduction of AgNO_3_ with citrate was used to synthesize AgNPs. The AgNPs with an average size of 30 nm were achieved through stepwise growth process using starter seeds. 

In general, carbon nanomaterials are functionalized by acid oxidation pretreatment to attach metal nanoparticles onto their surface. However, their electrical properties may be changed. The poly(vinylpyrrolidone) (PVP) grafting process causes much less structural damage and loss in the electrical conductivity of carbon nanomaterials than that for the conventional acid oxidation method [33]. Therefore, CB was surface-modified by a PVP grafting process. Firstly, 100 mg of CB was suspended in 15 mL of liquid vinylpyrrolidone containing 100 mg of benzoyl peroxide. The mixture was ultrasonicated in a ultrasonicator (Manufacture Expert, KX-1740QT, 120 W, 40 kHz, Ontario, CA, USA) for 10 min. Then, another 100 mg of benzoyl peroxide was added into the solution, which was further sonicated for another 10 min. This process was repeated five times until the CB was completely dispersed in the solution. During the ultrasonication process, CB was homogeneous surface functionalized and well dispersed in the vinylpyrrolidone solution. Anhydrous alcohol was used to dilute the obtained solution, which was then washed and vacuum filtered using filter membranes (pore size = 220 nm) with anhydrous alcohol to thoroughly remove physically absorbed polymers. Afterwards, the final products were dried at 75 °C in a drying oven (Suoyu Test Equipment Co., Ltd., Shanghai, China) to remove the residual solvent. 

### 2.3. Fabrication of CB/AgNPs Composite

To form CB/AgNPs composite, 100 mg of the PVP-grafted carbon black was dispersed in 50 mL of deionized water, and 100 mL of solution of obtained AgNPs with an average size of 30 nm was added drop by drop under vigorous stirring. Under this preparation condition, the mass ratio between CB and AgNPs in the present work was about 3:1. AgNPs were very effectively deposited on CB via the surface-grafted PVP moieties immediately. The CB/AgNPs composite was then collected by vacuum filtration, washed, and dried at 60 °C in a drying oven overnight. 

### 2.4. Fabrication of Strain Sensors

The TPU was dissolved in the solvent THF in a controlled environment with constant stirring and a controlled temperature of 60 °C. Subsequently, the obtained CB/AgNPs composite was mixed with the TPU in order to achieve a fixed mass fraction (CB/Ag-2, 5, 10, 15, 20, 30, 40 and 50 wt %) and left stirring for an additional 1 h to insure homogeneous dispersion of conductive fillers. Then the liquid mixture was wet-casted by applicator and the thickness of the wet film was 1 mm. After the evaporation of solvent, a sensitive film with a thickness of approximately 0.1 mm was produced. For further electrical and sensing tests, the film was cut into rectangle strips (3 cm × 1 cm) using a scalpel. As shown in Figure 1b, the sample displayed good stretchability when subjected to tensile strain. Then, two pieces of aluminum foil, acting as electrodes, were attached to the two ends of the strip by conductive silver paste (PELCO, TED PELLA), as shown in Figure 1c. To ensure the reliability of the sensors under tests, we encapsulated the overlapping parts between electrodes and the ends of rectangle strips using insulating tape.

### 2.5. Characterization

The morphologies of the AgNPs and CB/AgNPs composite were analyzed using a transmission electron microscope (TEM, TECNAI G2 F20, FEI company, Hillsboro, OR, USA) and field emission scanning electron microscope (FESEM, Hitachi S-4800, Hitachi, Tokyo, Japan). TEM samples were prepared by dispersing and diluting the AgNPs and CB/AgNPs composite in ethanol, and then casting suspensions onto copper TEM grids respectively. The stretching of the strain sensor and recycle experiment were all carried out using a universal testing machine (WDW-05, Jinan Shidaishijin Instruments Co., Ltd., Jinan, China) at a displacement rate of 10 mm/min. The universal testing machine was connected to the computer and controlled by the complied programs during the tests. The gauge length of the test samples was 10 mm. The resistance of the strain sensor was directly measured using the Agilent 34401A Digit Multimeter. The multimeter was connected with two electrodes of the sensor (Figure 1d) and a computer which was connected to the multimeter simultaneously showed the real-time electrical resistance during stretching. All experiments were performed in atmosphere at room temperature.

## 3. Results and Discussion

### 3.1. Morphology of Materials

**Figure 2:**
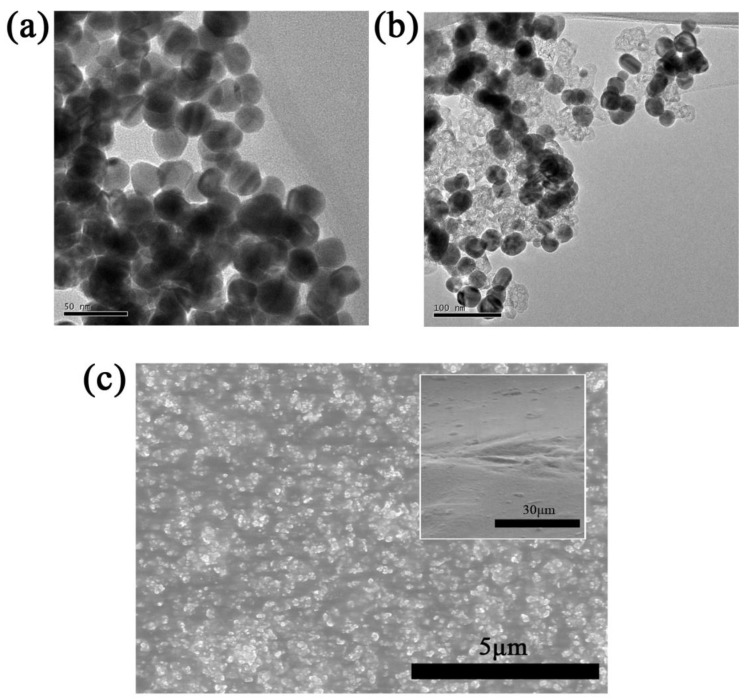
TEM images of (**a**) AgNPs and (**b**) CB/AgNPs composite; (**c**) SEM image of surface and cross-section (inset) morphology of TPU with CB/AgNPs composite.

The detailed microstructures of as-synthesized AgNPs and CB/AgNPs composite were firstly investigated. Figure 2a shows the TEM image of quasi-spherical AgNPs of ~30nm with a narrow size distribution. Moreover, the AgNPs were obtained without the formation of any other nano shape (i.e., nanorods, nanowires), indicating very uniform morphology. The TEM image of CB/AgNPs composite was also developed to demonstrate the assembling of CB and AgNPs. It can be observed that the AgNPs were well distributed on the surface of CB in Figure 2b. The structural damage is not observed, which means mild surface functionalization process of CB. The obtained CB/AgNPs composite was used as zero-dimension composite filler for fabrication of strain sensor. SEM analysis was utilized to investigate the morphology and dispersion of CB/AgNPs conductive networks in the TPU matrix. Figure 2c depicts SEM micrographs of the surface and cross-section (inset of Figure 2c) of the nanocomposite film which is composed of CB/Ag-30 and TPU. Generally, CB does not exist in the form of individual particles, but forms aggregates due to the van der Waals interaction. Thus, the fundamental structure unit of CB is aggregate, and the CB particles with AgNPs used in the present work represent aggregates. As can be seen in Figure 2c, the CB/AgNPs nanofillers are dispersed uniformly in the TPU matrix.

### 3.2. Electrically Conductive Properties

**Figure 3:**
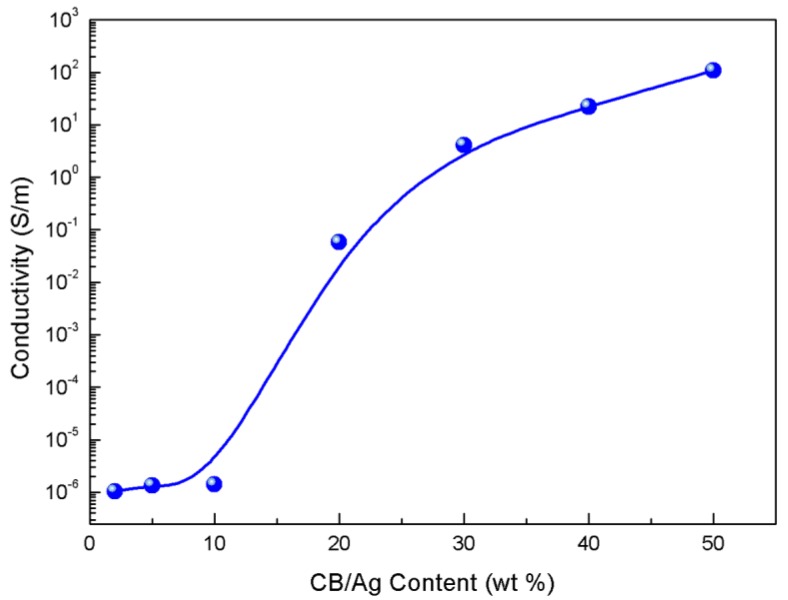
The electrical conductivity as a function of filler content for CB/AgNPs composite-based strain sensor.

To study the effect of the CB/AgNPs composite on the electrical properties of the strain sensor samples, CB/AgNPs composites with different filling levels were filled in TPU. The conductivity was calculated by using the formula: σ = L/RS, where σ is the conductivity, R is the resistance of the sample, S is the cross-section area of the strip, and L is the length. As anticipated, varying the content of the filler in TPU influenced the electrical characteristics of samples. It should be mentioned that compared to one-dimensional materials like CNTs, higher content of zero-dimension materials are needed for the construction of interlinked conductive networks because of their low aspect ratio [30]. Here, we chose 2, 5, 10, 15, 20, 30, 40, 50 wt % as different filling levels. Figure 3 displays the conductivity as a function of the filler concentration in TPU. As shown in Figure 3, a typical percolation transition behavior could be observed for CB/AgNPs-based samples. At low filling levels, such as 2, 5 and 10 wt %, the samples are almost insulated. This result indicates that the conductive networks are not formed due to small amounts of CB/AgNPs composite in insulated TPU. The conductivity enormously increases by several orders of magnitude when the filler loading ascends to a certain concentration range between 10 and 15 wt %, which means the conductive networks has been formed in the TPU matrix (the percolation zone). Compared to the percolation thresholds of other carbonaceous nanofillers, such as graphene nanoplatelets and CNTs in TPU [13,17,24,34], the relative high percolation threshold may be attributed to the low aspect ratio of CB/AgNPs and slight loss of electrical conductivity of CB through the surface modification process [33]. The conductivity of 20% and 30% filling level are 0.06 S/m and 4.13 S/m respectively. Then the conductivity curve tends to be gentle due to the denser network of CB/AgNPs composite with a relatively saturated concentration and reaches 22.33 S/m and 109.48 S/m at 40% and 50% filling levels respectively. The trend of as-obtained percolation curve is consistent with previous report [28], which indicates that the conductivity of the sensor becomes higher with increasing content of conductive material (more conductive paths). For resistive-type sensors, it is widely known that the high conductivity is favorable to design sensitive sensor materials driven by lower voltage [35,36]. If the conductivity of the sensor sample is too low, measuring its electrical resistance could be problematic [19]. Thus, the samples with 10% filling level are unacceptable for sensor applications. In addition, the CPCs near the percolation zone always exhibit higher sensitivity because the conductive networks are vulnerable to external stimuli [37]. In order to find the best balance between conductivity and sensitivity, the strain sensors with 20%, 30% and 40% content were then selected for strain sensing test.

### 3.3. Strain Sensing Characterization

Electrical characteristics are responsive to external mechanical stimulus when the CPCs-based strain sensors are subjected to tensile deformation, owing mainly to the evolution of conductive network. Normally, the slope of the relative change of resistance (ΔR/R_0_) versus strain curve reflects the gauge factor (GF), which is an essential parameter to assess the sensitivity of the strain sensor, defined as ΔR/(R_0_ε), where ΔR is the resistance variation, R_0_ is the initial resistance, and ε is the applied strain, respectively. The higher the GF value of a sensor, the more sensitive it is. In this work, the two ends of the strain sensor are clamped to insulating fixtures of the universal testing machine (Figure 1d) which is connected and controlled by computer; the multimeter is connected to electrodes to record the resistance variation caused by strains. The sensors are stretched automatically by the testing machine according to the designed program.

**Figure 4:**
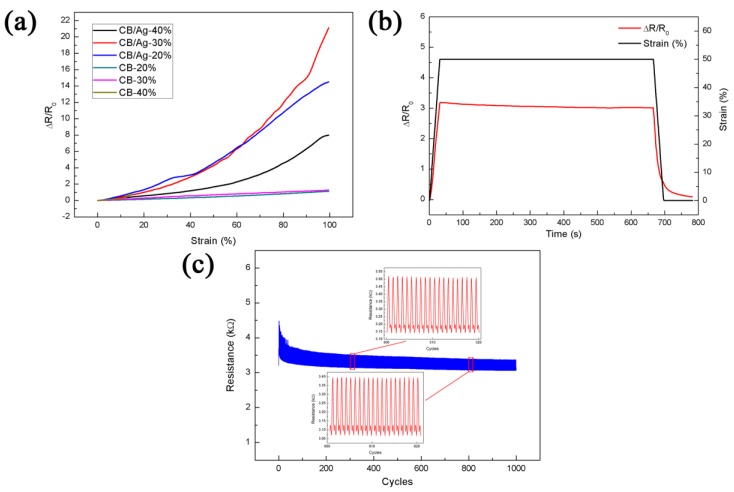
The properties of CB/AgNPs-based strain sensor. (**a**) The relative change in resistance under variable strains for three CB/AgNPs composite-based sensors with various filler concentrations (20%, 30%, 40%) and 20% CB-based strain sensor; (**b**) static characteristics with the 600 s hold time at a strain of 50%; (**c**) the repeatability test under a strain of 10% for 100 cycles. Inset is the enlarged view of the selected area.

Figure 4a compares the relative changes in resistance of CB/AgNPs composite-based sensors with three filling levels and also bare CB-based strain sensors with 20, 30, and 40 wt % filling levels (CB-20, CB-30, CB-40) upon strain. In order to ensure the reliability of testing data, we tested at least three samples for every different fabrication parameter (CB and CB/AgNPs at different filling levels). The samples under same fabrication parameter showed similar performance. It can be seen that the ΔR/R_0_ of all samples have a similar variation tendency. Among them, the responses of the samples based on bare CB shows smaller ΔR/R_0_ than other three samples throughout the stretching process, although rising monotonously with the increase of applied strain. The breakage of the CB-40 samples were observed when strain was increased to about 13%, and the samples were totally broken at ~15% strain. This may be caused by the decreased elasticity of the sample when the filling level of CB is exorbitant, leading to high brittleness. When it comes to the other two bare CB-based strain sensors, the ΔR/R_0_ of CB-30 (GF = 1.29 at 100% strain) is slightly higher than CB-20 (GF = 1.12 at 100% strain) over the process in our case although [38] selected CB-20 for their application. As for the CB/AgNPs composite-based sensors, when the filling level is 40%, the relative resistance change is slower than the other two filling levels as shown in Figure 4a. The reason is that as the filler concentration exceeds a certain level, more conductive paths in the CPCs can be constructed and the conductive network becomes more stable under tension, leading to the lower sensitivity. Therefore, the CB/Ag-40 may have fallen into the conductive zone in Figure 3 and not be sensitive enough. During the whole strain range, the sensor with CB/Ag-30 shows the best sensing property for strain sensing and has a GF of 21.12 at 100% strain, which is up to more than 18 times higher than the bare CB-20 strain sensor, although the resistance variation of the sensor with CB/Ag-20 is slightly higher in early stage of strain variation. In addition, although the linearity of CB/AgNPs-based sensors seems lower than bare CB-based sensors in Figure 4a, we find the CB/AgNPs-based sensors have three linear regions (0–40%, 40–70%, 70–100% strain) after the linearity fitting actually. The corresponding adjustive R-square of CB/Ag-30 in these three regions are 0.973, 0.981 and 0.965 respectively. The data shows that both CB/Ag-20 and CB/Ag-30 are in percolation zone and corresponding sensing performances are good for strain sensors. The reason is that compared to samples with higher filler contents like CB/Ag-40, conductive network constructed by less amount of fillers in the percolation zone can be destroyed easily, so higher sensitivities are obtained. However, to satisfy the practical application requirements as strain sensors, a sample with higher conductivity (CB/Ag-30) was selected for subsequent experiments to ensure the proper conductivity and sensitivity within a sufficient strain range. As can be seen in the data listed in Table 1, the working range and the GF is much higher, compared to previously reported work [10,39,40,41,42,43]. It is evident that the as-obtained sensor possesses better stretchability and sensitivity in the present work than in previous reports.

In order to observe the static characteristics of the sensor under static stretching state, a 50% strain was applied to the sensor and maintained for 10 min. Figure 4b shows a transient response to resistance. The ascending part of response curve is indicative of rapid resistance variation, which indicates good response property. Under a constant strain of 50%, a slight downward trend of resistance over the period of time was observed. This is generally detected in conventional filler-resin composite strain sensors, but the variation of ΔR/R_0_ is less than 5% throughout the process at present work, which means good static stability. Besides, no remarkable resistance fluctuation was observed. The response of the sensor decreased immediately when the strain began to decline. When the strain was reduced to 0%, the sensor still needed some time (~60 s) to recover to the resistance which is very close to its initial resistance. The recovery time is different from the 100 s recovery time of the polymer elastomer with CB filler [18].

Repeatability is also a crucial feature when flexible strain sensors are used as wearable devices during repeated stretching and releasing cycles. The dynamic strain sensing behavior was thus investigated to evaluate the repeatability of the CB/AgNPs-based strain sensor. Figure 4c shows the dynamic characteristic of resistance variation under 1000 stretch-and-release cycles for a strain of 10%. It is clearly shown that the resistance of the sensor increases during stretching periods and decreases during releasing periods, and the relative change of resistance becomes steady after a few cycles and exhibits good repeatability. In other words, the strain sensing response has a downward drift for the initial several cycles and then maintains a stabilized tendency. This is normal for a resistive-type strain or stress sensor and can be attributed to the permanent damage in the conductive networks and hysteresis effect resulting from the viscoelastic properties of TPU [37,44]. As the elastomeric materials exhibit creep behavior and there is a competition between network breakup and rebuilding during loading and unloading, a gradual drop of ΔR/R_0_ was observed. Similar observations have been reported in literature [17,24]. As for the resistance of the sensor, it increased after the first cycle and then gradually reduced by 2.4% of the initial resistance values after 1000 cycles. This is because the conductive networks were rearranged along with the motion of the TPU molecular chains, namely, the conductive nanofillers are reorganized in TPU matrix under repeated stretch-and-release cycles, which is consistent with the results for TPU with graphene nanoplatelets filler [17]. Besides, both the breakdown and reconstruction of electrically conductive networks coexist during the cycles, and they gradually approach an equilibrium state after several cycles [28]. The inset in Figure 4c shows the stable reproducibility extracted from the 300 to 320 and 800 to 820 cycles respectively. 

### 3.4. Sensing Mechanism

The sensing mechanism of the CB/AgNPs composite-based strain sensors can be ascribed to the following two mechanisms. Disconnection mechanism should be considered firstly. In conductive films made of nanomaterial conductive network, electrons can pass through overlapped nanomaterials within the percolation network [20]. Stretching of the sensors causes some connected nanomaterials to lose their overlapped area and electrical connection, and consequently, increases the electrical resistance. Secondly, when the overlapped two parts separate, the change in resistance of the strain sensor is conducted by the particles tunneling effect. Crossing of electrons through a nonconductive barrier is called tunneling. Electrons can tunnel through closely spaced adjacent nanomaterials [10]. The tunneling current can be formed (R_tun_) when the distance between two neighboring nanoparticles is in the range of the cut-off distance, because the electrons can tunnel through the polymer matrix and form a quantum conductive junction [45,46]. Furthermore, if the distance between nanoparticles exceeds the cut-off distance, there will be no electron flow pass through such nanoparticles and the corresponding electrical path is considered to be disconnected (R_disc_).

**Figure 5:**
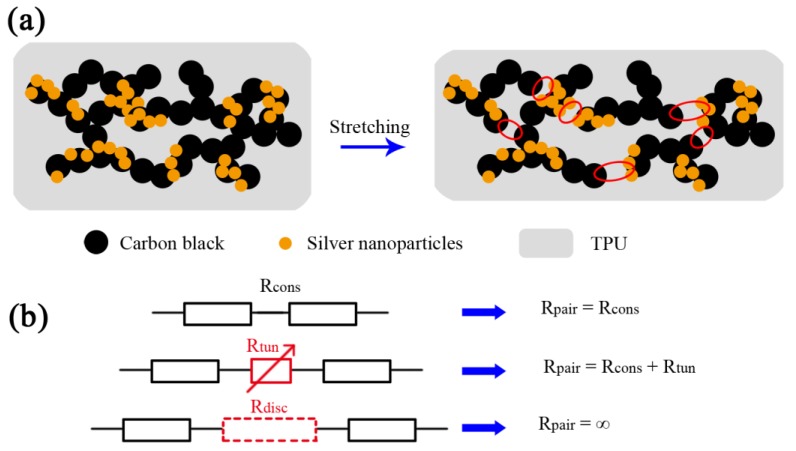
(**a**) Morphology evolution of CB/AgNPs-based strain sensor during stretching (the red circles represent junction resistance between nanoparticles); (**b**) different electrical models of interconnections between two adjacent NPs.

The structure models of the CB/AgNPs composite-filled TPU without and under strain are shown in Figure 5a. The junction resistance between every pair of nanoparticles in this composite (red circles in Figure 5a) can be seen as a variable resistor containing constant resistance of composite (R_cons_, it will not change during the deformation), R_tun_ and R_disc_ in strain sensing. This is because with the increase of applied strain, the resistance will change due to the variation of distance of neighboring nanoparticles. The equivalent electrical circuit for each configuration is shown in Figure 5b. The mechanism is also applicable for CB-based sensors. The addition of AgNPs contributes to form three different junctions in the composite, including the junctions between AgNPs, CBs and AgNPs and CBs [47], instead of single junction between CBs in CB-based strain sensors. These different junctions provide more variable resistors than single junction during the deformation. As a result, when the strain is applied on the CB/AgNPs composite-based sensor, the variable junction resistance will increase more remarkably. There is a good agreement between the proposed sensing mechanism and experimental results shown in Figure 4a. Therefore, the CB/AgNPs composite-based sensor shows higher sensitivity compared with the bare CB-based sensor. 

### 3.5. Applications

**Figure 6:**
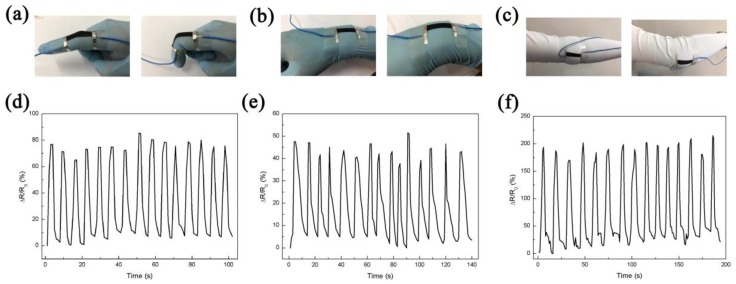
Photos of the strain sensor attached on (**a**) finger; (**b**) wrist and (**c**) elbow; ΔR/R_0_ vs. time curves under operations of (**d**) finger bending; (**e**) wrist rotation and (**f**) elbow flexion.

Considering the good sensitivity and stretchability, the CB/AgNPs composite-based strain sensor has the potential to monitor human motions (Figure 6a–c). In order to demonstrate the capability of the sensor for joint movements detection, the strain sensor was first attached to a latex glove on the position of the forefinger joint. The adhesive tape was used to fix both ends of the sensor for a stable connection, as shown in Figure 6a. Obvious relative change of resistance can be observed when bending the index finger. Figure 6d illustrates ΔR/R_0_ versus time curve for cyclic bending and straightening motions of the forefinger. The ΔR/R_0_ (~0.8) for motion detection of the index finger has been increased when compared with the work reported before (under 0.5) [10,27], which is competitive in similar applications. The sensor can also test the wrist rotation (Figure 6b) when it was mounted on the wrist. The response varied with respect to the degree of wrist bending, as shown in Figure 6e. In addition, the sensor was fixed on the elbow joint (Figure 6c) for large-scale movement detection in real time, which is rarely reported for those detecting applications based on strain sensors with limited stretchability [48,49]. The resistance increased simultaneously with the bending of the elbow and the larger ΔR/R_0_ was recorded when the elbow was fully bending (Figure 6f). In addition, we tested the resistance of the CB/Ag-30 sensor under different temperature (from room temperature to 60 °C). We used a flat heating plate which is controlled by a digital temperature controller to heat the sensor in this test. Although the resistance slightly ascends with increased temperature, the variation was only 4.86% compared to the initial resistance (3.09 kΩ) throughout the heating process. Moreover, the resistance was stable at each temperature testing point. Since the changing range of the ambient temperature is generally from room temperature to 40 °C, the resistance variation is 1.13% under this temperature range, and the change of temperature on the body or textile is small for normal usage, thus the temperature will not have a remarkable influence on the sensor. Overall, the results presented that the CB/AgNPs composite-based strain sensor might be potentially applied as a promising wearable and flexible strain sensor for monitoring of various human joint motions in real time.

## 4. Conclusions

In summary, we present a new type of strain sensor based on carbon black/silver nanoparticles composite via a simple surface modification, deposition, combination and casting process. Due to the addition of AgNPs in CB particle-to-particle conductive networks, the number of junctions increases markedly so that the variation of resistance can change significantly under stretching, leading to higher sensitivity than bare CB-based strain sensor. Moreover, the electrical conductivity and sensing performance can be simply controlled by tuning the composite’s filling level in TPU. The static and dynamic characteristics of resistance change of the sensors under external stress were measured. The developed strain sensor exhibited high and stable sensitivity in a wide range of strain, and the sensing mechanism derived from resistance model using junction identification was further discussed in detail. The sensor also showed promising capability in the monitoring of various human motions such as finger bending, wrist rotation and elbow flexion. We believe that the reported strain sensor demonstrates its high potential for the applications in wearable devices for the human body.

## Figures and Tables

**Table 1:** Comparison of working range and GF of previous reported strain sensors based on different sensitive materials.

Material	Working Range	Gauge Factor	Year	Reference
CB/AgNPs composite/TPU	100%	21.12 at 100% strain	–	This work
TPU/MWCNTs/NFC	50%	3	2017	[39]
Ag nanowires/PDMS	70%	2~14	2014	[10]
Ag nanoparticles/PDMS	20%	2.05 at 20% strain	2014	[40]
TPU/SBS/MWCNTs	50%	1.8	2014	[41]
PPy/PU	300%	2.32 at 50% strain	2013	[42]
TPU/CNT/TPU yarn	10%	4	2013	[43]
